# Identification of aberrantly expressed lncRNAs and ceRNA networks in multiple myeloma: a combined high-throughput sequencing and microarray analysis

**DOI:** 10.3389/fonc.2023.1160342

**Published:** 2023-06-05

**Authors:** Min-Qiu Lu, Yu-Qin He, Yin Wu, Hui-Xing Zhou, Yuan Jian, Wen Gao, Li Bao, Wen-Ming Chen

**Affiliations:** ^1^ Department of Hematology, Beijing Jishuitan Hospital, Beijing, China; ^2^ Department of Emergency, Beijing Chao-Yang Hospital, Capital Medical University, Beijing, China; ^3^ Department of Hematology, Beijing Chao-Yang Hospital, Capital Medical University, Beijing, China

**Keywords:** long noncoding RNAs, multiple myeloma, genomics, high-throughput sequencing, microarray

## Abstract

**Background:**

This study aimed to explore the potential effects of long non-coding RNAs (lncRNAs) in multiple myeloma (MM) patients using two detection methods: high-throughput sequencing and microarray.

**Methods:**

In this study, lncRNAs were detected in 20 newly diagnosed MM patients, with 10 patients analyzed by whole transcriptome-specific RNA sequencing and 10 patients analyzed by microarray (Affymetrix Human Clariom D). The expression levels of lncRNAs, microRNAs, and messenger RNAs (mRNAs) were analyzed, and the differentially expressed lncRNAs identified by both methods were selected. The significant differentially expressed lncRNAs were further validated using PCR.

**Results:**

This study established the aberrant expression of certain lncRNAs involved in the occurrence of MM, with AC007278.2 and FAM157C showing the most significant differences. The top 5 common pathways identified by the Kyoto Encyclopedia of Genes and Genomes (KEGG) analysis were the chemokine signaling pathway, inflammatory mediator regulation, Th17 cell differentiation, apoptosis, and NF-kappa B signaling pathway. Furthermore, three microRNAs (miRNAs) (miR-4772-3p, miR-617, and miR-618) were found to constitute competing endogenous RNA (ceRNA) networks in both sequencing and microarray analyses.

**Conclusions:**

By the combination analysis, our understanding of lncRNAs in MM will be increased significantly. More overlapping differentially expressed lncRNAs were found to predict therapeutic targets precisely.

## 1 Introduction

Multiple myeloma (MM) is a plasma cell neoplasm characterized by the clonal proliferation of malignant plasma cells in the bone marrow microenvironment (1). Proteasome inhibitors, immunomodulatory drugs, and CD38-targeting antibodies have been extending survival (2, 3). Despite the recent advances in clinical and experimental oncology, many patients will relapse and die from MM. Disease progression and subsequent relapses are characterized by increasingly resistant disease and subclonal evolution. Risk stratification based on disease-specific is important for prognosis and the treatment strategy (4). Many current research strategies were used to guide the therapy and refine the immunotherapeutic approaches (5, 6).

Accurate prognostic evaluation and therapeutic targets of multiple myeloma depend on gene detection. Thus, a detailed understanding of the mechanisms is very important. Most long non-coding RNAs (lncRNAs) regulate tumor proto and suppressor oncogene pathways, as transcribers record regulatory factors (7, 8). In addition, epigenetic modification and interaction with RNA-binding proteins had also been confirmed to be the main mechanisms and functions of lncRNAs (9). Through a competing endogenous RNA (ceRNA) network, lncRNAs regulate the function of microRNA (miRNA) as a miRNA sponge (10, 11). LncRNAs are involved in the occurrence and development of MM. The action modes of different lncRNAs are very different, and upregulated or downregulated of lncRNAs can lead to various carcinogenic tendencies in plasma cells (12, 13). The aberrant expression of lncRNAs in more aggressive stages of MM has been found (14). Ronchetti et al. (14) provided a special view of the expression of lncRNA in MM. In previous studies, through high-throughput sequencing, some lncRNAs were found abnormally expressed in newly diagnosed MM, including maternally expressed gene 3 (MEG3), colon cancer-associated transcript 1 (CCAT1), and coiled-coil domain-containing 26 (CCDC26) (15–17). In addition to known lncRNAs, some unpublished lncRNAs were found, and new RNAs related to MM pathogenic genes were identified, providing new targeted therapies for MM.

RNA-Seq can be applied to investigate different populations of RNA, including messenger RNA (mRNA), circular RNA (circRNA), and lncRNA. High-throughput sequencing technology can be called a milestone in the development of sequencing technology. In addition to high-throughput sequencing, gene microarray has also become an important method of gene detection, and gene chip has the analytical ability to fully display the genetic spectrum of target samples through the mechanism of hybridization (18). Compared with sequencing, gene microarray is not possible to discover novel genes, but it will focus on lncRNAs that have been found to play a role in disease. Affymetrix Human Clariom D Array is a new generation of gene microarray chips, which detects mRNA, lncRNA, miRNA, and circRNA information of known sequences. Whether sequencing or gene microarray, software tools can perform hierarchical clustering, Gene Ontology (GO) enrichment analysis, pathway enrichment analysis, co-expression network (lncRNA–circRNA–mRNA), lncRNA target pathway network, and ceRNA network (lncRNA–miRNA–mRNA or circRNA–miRNA–mRNA). Through the analysis of this information, many unknown functions of lncRNA in MM can be inferred.

In our study, we conducted a comparative analysis of high-throughput sequencing data and gene chip data for lncRNAs. The aim was to identify overlapping differentially expressed genes, thereby allowing for the identification of more precise target genes that are significantly associated with the occurrence and progression of MM.

## 2 Materials and methods

### 2.1 Patients and cell samples of bone marrow

The plasma cells of bone marrow were collected from 20 MM patients, and normal individuals were taken as the control. The patients met the updated diagnostic criteria of the International Myeloma Working Group (IMWG) for MM (19). Inclusion criteria: 1) p reviously untreated patients diagnosed with multiple myeloma according to the IMWG criteria. 2) According to IMWG standard disease, there are evaluable blood and urine M protein indexes (serum m-protein ≥1 g/dl (≥10 g/L) and serum m-protein ≥0.5 g/dl (≥5 g/L) in IgA, IgD, IgE, or IgM multiple myeloma subjects; or urine m-protein levels ≥200 mg/24 h; or light chain, no measurable lesions in serum or urine, serum immunoglobulin free light chain (FLC) ≥10 mg/dl and serum immunoglobulin Kappa Lambda FLC ratio abnormalities). There may be a measurable extramedullary mass. Exclusion criteria: 1) p atients did not meet the MM diagnostic criteria updated by the International Myeloma Working Group, 2) p atients with relapsed and refractory multiple myeloma, 3) MM combined with other tumors, 4) MM prepared for autologous stem cell transplantation, and 5) s olitary plasmacytoma. This study met the standards of medical ethics and was approved by the hospital ethics committee. All participants provided signed informed consent, and this study was approved by the institutional ethical review board of the Chao-Yang Hospital, Capital Medical University (ethical approval code 2016-science-84, 2019-science-118). The median age of the patients was 63 (53–74) in the sequencing group and 61 (47-82) in the chip group.

Mononuclear cells were extracted from 5 ml of bone marrow. Plasma cells were sorted by CD138 magnetic beads. Total RNA was extracted using a TRIzol reagent. Non-coding RNAs of 10 patients were detected by sequencing and those of 10 patients by gene chip. Total RNA was purified using miRNeasy Serum/Plasma kit (Qiagen, Hilden, Germany).

### 2.2 RNA sequencing and identification of lncRNAs by high-throughput sequencing

Raw sequencing data were filtered out. The clean data were obtained after filtering. To ensure the quality of the data used for information analysis, the raw data (raw reads) obtained from sequencing were initially filtered using in-house programs. The filtering conditions included the following: 1) removal of reads contaminated with adapter sequences (reads with adapter contamination > 5 bp were removed; for paired-end sequencing, if one end was contaminated, both ends of the read were removed); 2) removal of low-quality reads (reads with base quality values of Q ≤ 19 accounting for > 15% of the total bases; for paired-end sequencing, if one end was of low quality, both ends of the read were removed); 3) removal of reads with an N ratio > 5% (for paired-end sequencing, if one end had an N ratio > 5%, both ends of the read were removed). The filtered data, referred to as clean data, were subjected to quality and quantity analyses, including Q30 calculation, data volume calculation, and base composition analysis. Reference genomes were downloaded from Ensembl (http://www.ensembl.org/index.html), and the clean data were mapped to the reference genome through HISAT2 (http://ccb.jhu.edu/software/hisat2/index.shtml) (20–23). FPKM (Fragments Per Kilobase Million Mapped Reads) were calculated for the gene expression of all samples. DESeq (http://www.bioconductor.org/packages/release/bioc/html/DESeq.html) was used for differential expression analysis (21, 22). The mRNAs with Spearman’s correlation coefficient (p ≥ 0.9) were selected as the *trans*-targets, and mRNAs with a distance of less than 50 kb were selected as the *cis*-targets. To identify potential lncRNAs from the sequencing data, a series of steps were employed. First, transcripts with coding potential were filtered out based on transcript length, exon number, and other relevant criteria. Transcripts with low read coverage were also eliminated across all samples, as well as known coding RNAs and non-coding RNAs such as rRNAs, tRNAs, snoRNAs, and snRNAs. Next, the class_code information from gffcompare was used to categorize the remaining transcripts into lincRNA, intronic lncRNA, or anti-sense lncRNA. Finally, a comprehensive analysis was performed using various coding potential analysis tools, including CNCI, CPC, PFAM protein domain analysis, and CPAT, to confirm the non-coding nature of the remaining transcripts. These transcripts were then identified as novel lncRNAs.

### 2.3 Identification of DE lncRNAs for the microarray

The human Clariom™ D array (Affymetrix, Santa Clara, CA, USA) was used to analyze the expression of lncRNAs, miRNAs, and mRNAs. The ceRNA network was generated using GeneChip Command Console Software (AGCC version 4.0; Affymetrix) (24). One-Cycle Target Labeling and Control Reagents (Affymetrix, Santa Clara, CA, USA) were used for cDNA synthesis, and cRNA was generated using GeneChip^®^ IVT Labeling Kit (Affymetrix). cRNA was fragmented and then hybridized to Affymetrix Human Clariom D array. After being washed and stained in the Affymetrix Fluidics Station 450, GeneChips were scanned by using Affymetrix GeneChip Command Console (AGCC version 4.0) installed in GeneChip^®^ Scanner 3000 7G. The original data (CEL.files) were analyzed by Robust Multichip Analysis (RMA) algorithm. Summarizing Affymetrix default analysis settings and global scaling as a quantile normalization method, Log2 RMA signal intensity was obtained.

Hierarchical clustering is a powerful and visualized tool for analyzing the status of all genomic datasets. The matrix distance between the gene expression data was calculated by Cluster_Treeview software (Palo Alto, CA, USA). The statistical method was a *t*-test for the mean of two samples, the differentially expressed genes were selected when the p- value < 0.05 and the threshold of fold change (FC) value ≥2.0. The heatmap of all differential genes and all samples was illustrated in visualization by the Tree view (25, 26).

### 2.4 Enrichment analysis

GO (http://geneontology.org/) provides a standard vocabulary of gene function, including molecular function, cell component, and biological process. Kyoto Encyclopedia of Genes and Genomes (KEGG; http://www.kegg.jp/) contains the molecular interaction and reaction networks of the genes. Functional enrichment analysis based on hypergeometric distribution, the significantly enriched GO terms, and KEGG pathways are identified.

### 2.5 CeRNA analysis

CeRNA is a deep analysis of mRNA, lncRNA, circRNA, and microRNA (27, 28). Target genes of the miRNAs were predicted by MiRanda, PITA, and TargetScan, and the target genes were the cumulative outcome of the prediction using at least two programs.

Pearson’s correlation coefficient (PCC) was calculated according to the expression of mRNA and lncRNA. The number and binding score of miRNA response elements (MREs) can evaluate the ability of ceRNA molecules (27, 28).

### 2.6 Quantitative real-time reverse transcriptase–PCR

The expression of two downregulated overlapping differentially expressed (DE) lncRNAs in MM patients was examined using high-throughput sequencing and microarray using the quantitative reverse transcriptase– polymerase chain reaction (RT-PCR). Total RNA was isolated by RNA Extraction Kit DNase I (GenePool, Beijing, China; Cat# GPQ1801). The cDNA was synthesized using lncRNA cDNA Synthesis Kit with GenePool (Cat# GPQ1806), and quantitative PCR was performed on the Line Gene 9600 Plus (Bioer Technology, Zhejiang, China). Briefly, PCRs were performed in a triplicate mixture (20 µl) containing dNTP Mix 2.5 mM each 4 µl, Primer Mix 2 µl, RNA Template 2 µl, 5× RT Buffer 4 µl, DTT 0.1M 2 μl, HiFiScript 200 U/μl, RNase-Free Water up to 20 μl. GADPH was used as an internal control. After amplification, a melting curve analysis was performed to confirm reaction specificity. The amplification procedure was as follows: 95°C for 35 s, (95°C for 5 s, 60°C for 30 s) × 45 cycles, and conduct melting curve analysis simultaneously at 60-95°C. The upstream primer of AC007278.2 is CCATTCATCCACAACGCAAGG, and the downstream primer is TGGCAGACACAGAGTATCTTCAC. The upstream primer of FAM157C is TCAGGACCTACTCGGGAGAC, and the downstream primer is CTGAGGAGCTGAGGAGAAGGA.

## 3 Results

### 3.1 Clinical characteristics of patients with MM in the two groups

The clinical and genetic characteristics of the 20 patients with MM in the two groups were recorded and shown in **Table 1**. The high-risk MM patients in the sequencing group and gene chip group were 70% and 80%, respectively.

**Table 1:** Clinical characteristics of patients with MM.

Characteristics	Patient number of sequencing (*n* = 10)	Patient number of gene chip detection (*n* = 10)
Age (year) ≧60	6 (60%)	6 (60%)
Sex		
Male	6 (60%)	8 (80%)
Female	4 (40%)	2 (20%)
Immunoglobulin type		
IgA	2 (20%)	3 (30%)
IgG	7 (70%)	1 (10%)
Light chain type	1 (10%)	4 (40%)
International Staging System (ISS) at diagnosis		
Durie-Salmon (DS) stage		
I	1 (10%)	1 (10%)
II	2 (20%)	1 (10%)
III	7 (70%)	8 (80%)
ISS stage		
I	1 (10%)	2 (20%)
II	2 (20%)	2 (20%)
III	7 (70%)	6 (60%)
Complicated with nephropathy	2 (20%)	4 (40%)
Complicated with extramedullary lesions	1 (10%)	0 (0%)
Cytogenetics/FISH		
Standard risk	3 (30%)	2 (20%)
High risk: del(17p), t(4;14), t(14;16), 1q21	7 (70%)	8 (80%)

MM, multiple myeloma; FISH, fluorescence in situ hybridization.

### 3.2 DE lncRNAs in sequencing group

**Figure 1:**
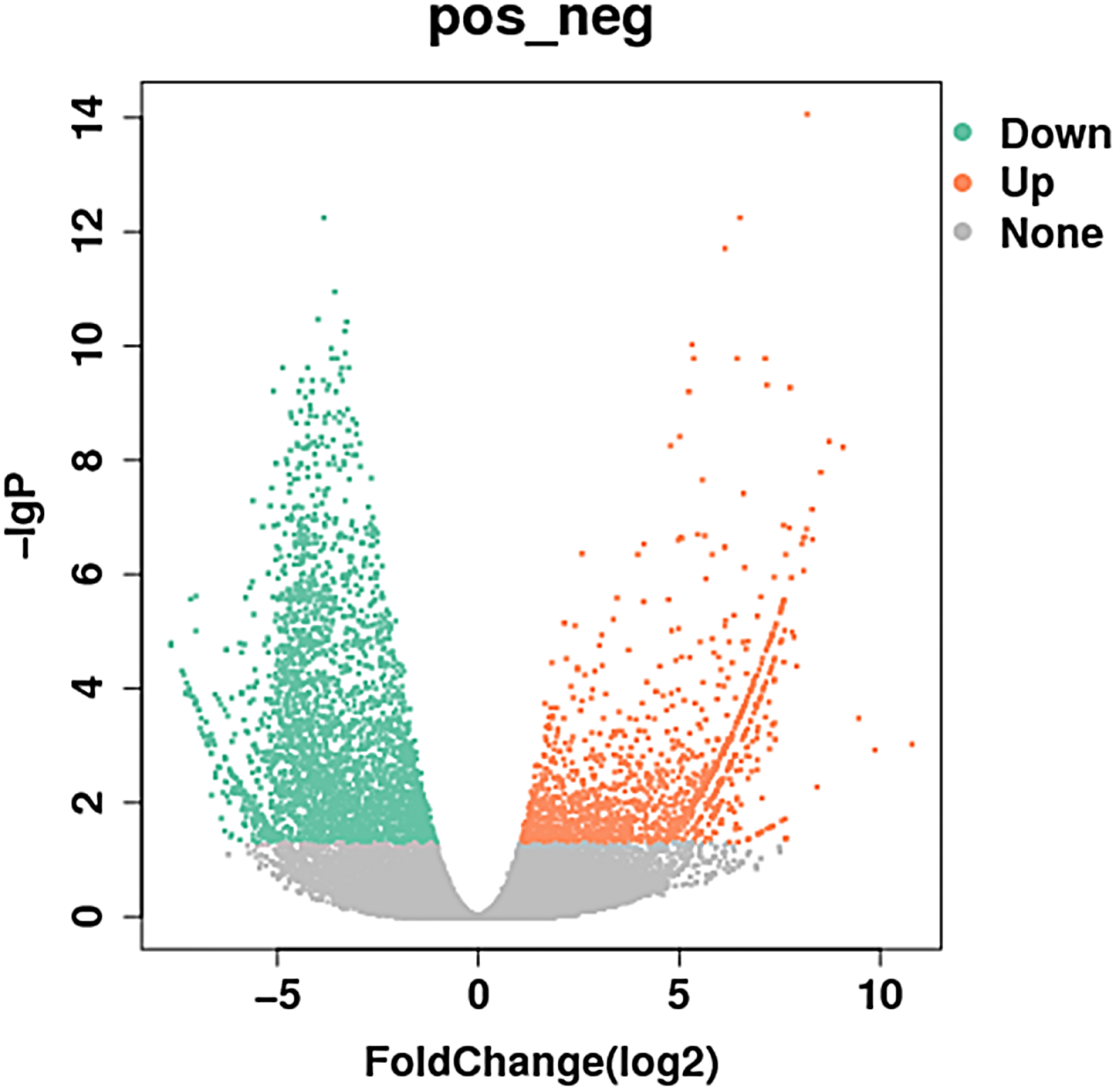
Volcanic map of differentially expressed known lncRNAs in the sequencing group: the abscissa is the expression of multiple changes in different experimental groups, the ordinate is the statistical significance of the expression change, red and green respectively represent differentially expressed upregulated and downregulated genes, and gray represents non- differentially expressed genes (910 upregulated and 649 downregulated). Different colors represent different classifications. lncRNAs, long non-coding RNAs.

**Figure 2:**
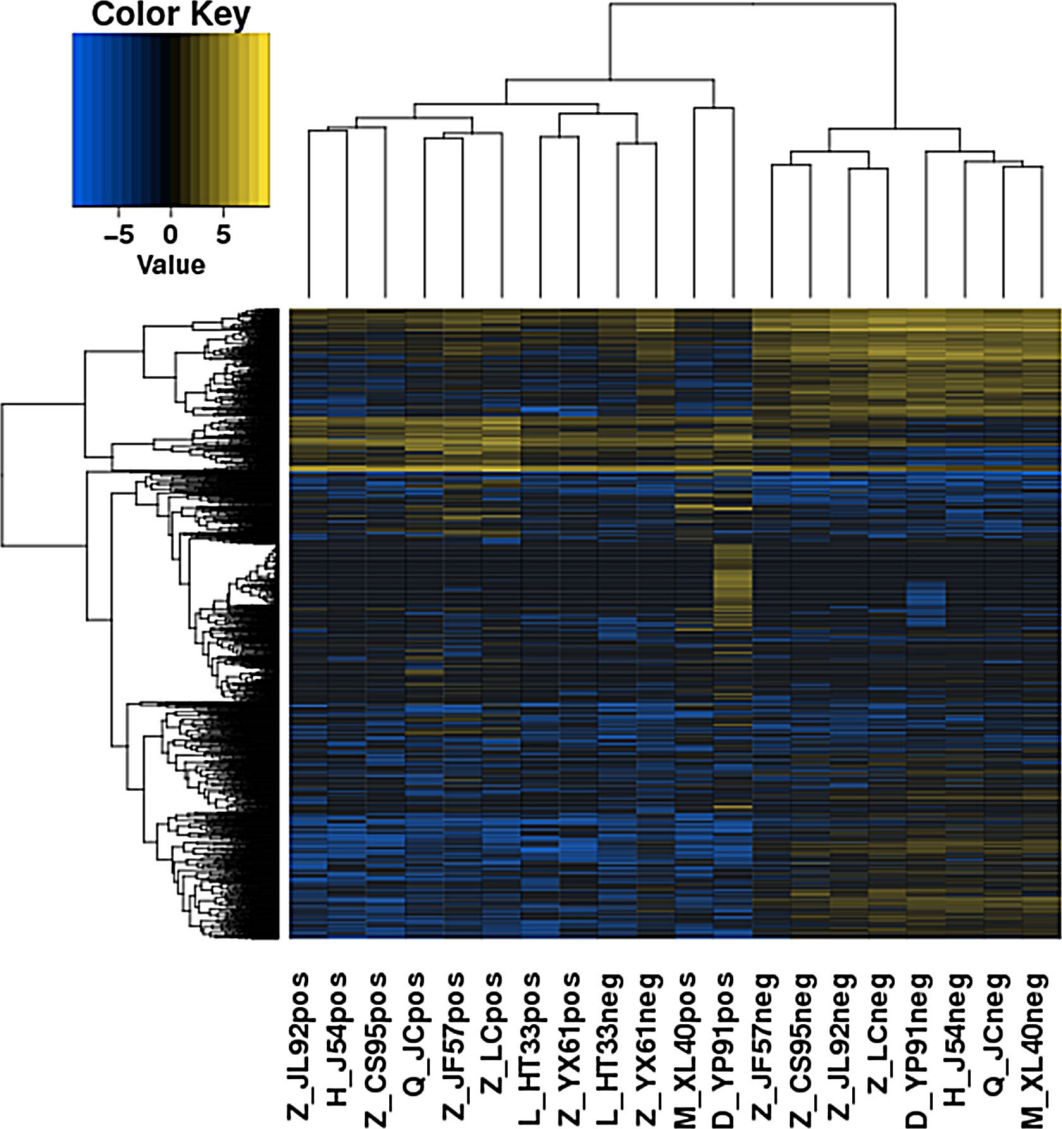
Heatmaps of DE lncRNAs in the sequencing group. The various colors represent different expression levels. Blue color indicates the lower expression level, and yellow color indicates higher expression levels. DE lncRNAs, differentially expressed long non-coding RNAs.

Compared to the control group, MM cells exhibited a significant difference in the expression of lncRNAs, with a total of 4,419 DE lncRNAs identified. Among these DE lncRNAs, 1,559 were known lncRNAs that showed differential expression, with 910 upregulated and 649 downregulated. The volcanic map of differentially expressed known lncRNAs in the sequencing group is shown in **Figure 1**. The clustering results of the known lncRNAs are shown in **Figure 2**. The top 20 DE known lncRNAs are shown in **Table 2**.

**Table 2:** Analysis of top 20 known DE lncRNAs in sequencing group.

Gene ID	LncRNA	FoldChange	Log2 fold change	pval	padj	Up/down
ENSG00000236525	AC007278.2	0.031389875	−4.993556912	3.62835E−10	1.3377E−07	Down
ENSG00000234389	AC007278.3	0.0303423	−5.042525722	2.5526E−09	5.6236E−07	Down
ENSG00000227039	ITGB2-AS1	0.037552813	−4.734935192	1.26053E−08	2.1085E−06	Down
ENSG00000268833	AC011513.4	0.030671627	−5.026951498	3.94915E−08	5.09591E−06	Down
ENSG00000254141	RP11-642D21.1	82.5077377	6.366457519	4.11248E−08	5.26903E−06	Up
ENSG00000255197	RP11-750H9.5	0.051259714	−4.286030762	4.2015E−08	5.35775E−06	Down
ENSG00000273036	FAM95C	0.036722734	−4.767182706	4.23594E−08	5.37638E−06	Down
ENSG00000237298	TTN-AS1	0.195507254	−2.354705961	4.6911E−08	5.84457E−06	Down
ENSG00000268734	CTB-61M7.2	0.04281355	−4.545788725	4.7043E−08	5.84758E−06	Down
ENSG00000185433	LINC00158	28.18500071	4.816855698	8.90195E−08	9.74211E−06	Up
ENSG00000261997	RP11-212I21.4	0.053291433	−4.229952555	1.17233E−07	1.21251E−05	Down
ENSG00000254281	KB-1507C5.4	0.074209599	−3.752250373	1.47235E−07	1.45612E−05	Down
ENSG00000231204	AC011752.1	105.1655094	6.716517818	1.55249E−07	1.50517E−05	Up
ENSG00000260528	FAM157C	0.101704459	−3.297545156	1.64661E−07	1.56836E−05	Down
ENSG00000226091	LINC00937	0.059305153	−4.075698727	1.9264E−07	1.77115E−05	Down
ENSG00000275527	CTD-3154N5.2	0.072151543	−3.792825934	3.04896E−07	2.56586E−05	Down
ENSG00000274173	RP4-568C11.4	0.046124253	−4.438330658	4.90195E−07	3.83357E−05	Down
ENSG00000259652	RP11-797A18.3	0.030860671	−5.018086763	5.00197E−07	3.89492E−05	Down
ENSG00000180539	C9orf139	0.064676753	−3.950608935	8.13188E−07	5.86838E−05	Down

DE lncRNAs, differentially expressed long non-coding RNAs.

### 3.3 D ifferentially expressed lncRNAs in gene microarray group

**Figure 3:**
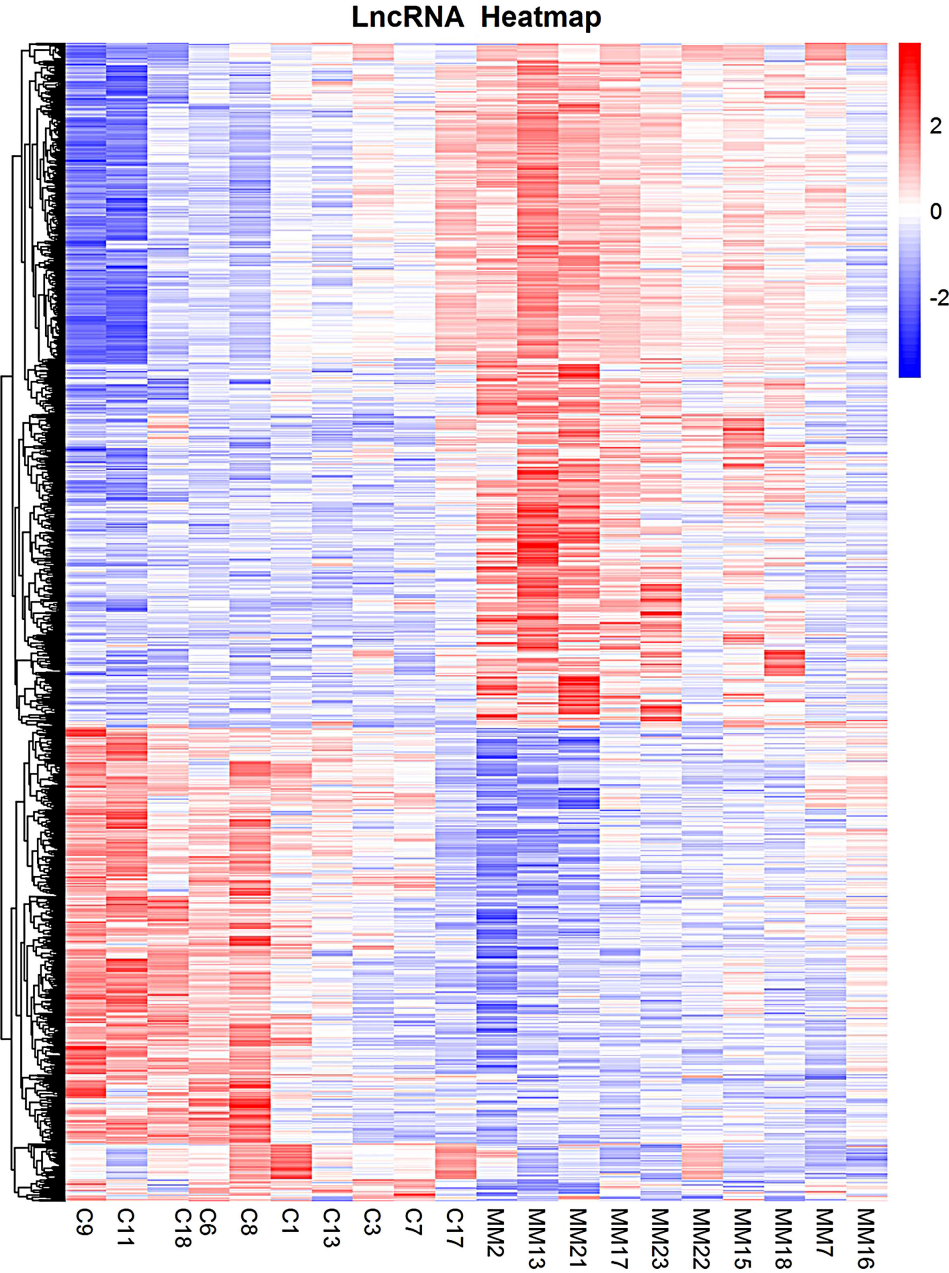
Heatmaps of DE lncRNAs in gene microarray group. Each column represents specimens with different serial numbers. C represents the control group, and MM represents the experimental group. The red box indicates the upregulated lncRNA molecule. The blue box indicates the down regulated lncRNA molecule. DE lncRNAs, differentially expressed long non-coding RNAs.

A total of 1,263 DE lncRNAs were identified in microarray (748 upregulated and 515 downregulated). The clustering results of the lncRNAs of the samples in the gene microarray group according to the expression of differentially expressed genes are shown in **Figure 3**.

### 3.4 Overlapping DE lncRNA in sequencing group and gene microarray group

**Figure 4:**
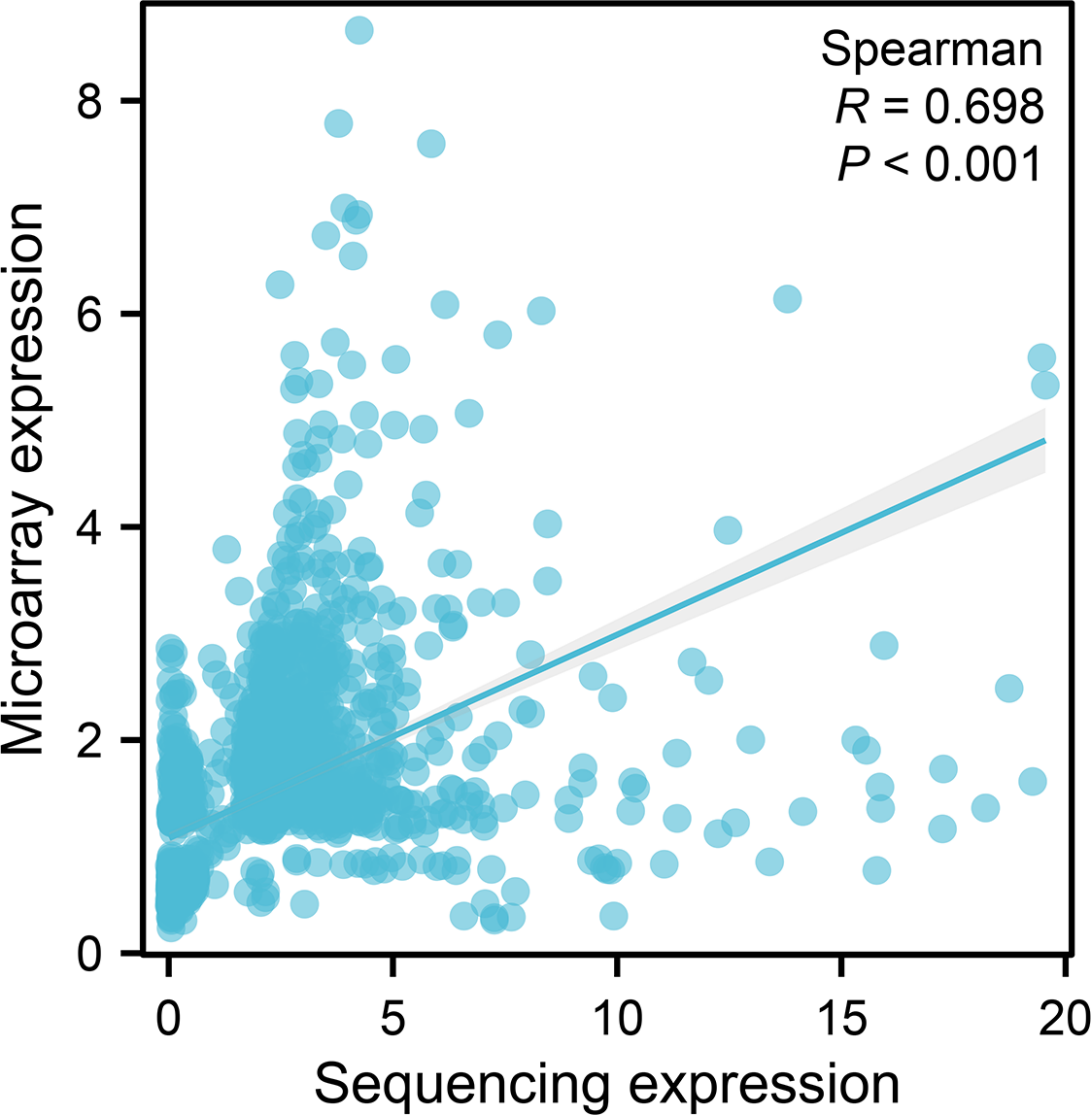
A scatter plot of the expression of the microarray group versus the sequencing group of the common molecules. The horizontal coordinates represent the fold-change expression of sequencing, and the vertical coordinates represent the fold-change expression of microarray. The dots indicate the common molecules between differentially expressed (p < 0.05) molecules based on sequencing and microarray. The line indicates regression line. p-Value and R-value were calculated by Spearman’s correlation.

A total of 64 overlapping DE lncRNAs were detected by the two methods (26 genes were upregulated, as shown in **Table 3**, and 38 were downregulated, as shown in **Table 4**). The correlation between the two methods is shown in **Figure 4** (R = 0.698, p < 0.001). Among the 26 upregulated genes, IFNG-AS1, AC093818.1, AF127936.5, and OLMALINC were expressed in different diseases, especially IFNG-AS1, which has been reported in many malignant tumors (29–31). Among the down regulated genes, DLEU2, LINC01270, LINC01270, GK-IT1, LINC01001, DLEU2, CHRM3-AS2, AC007278.2, PLBD1-AS1, FAM157C, KCNJ2-AS1, and LINC01127 were abnormally expressed in other diseases in previous studies (32, 33). AC007278.2 and FAM157C were identified as the most significantly differentially expressed lncRNAs between the MM cells and the control group.

**Table 3:** Overlapping upregulated DE lncRNAs in sequencing group and gene microarray group.

LncRNA	High-throughput sequencing			Gene chip		
	FoldChange	pval	Up/down	FoldChange	Regulation	p-Value
AC093818.1	3.006081272	0.004855534	Up	1.58704477	Up	0.00608811
RP11-70P17.1	3.732231545	0.021127895	Up	1.81088483	Up	0.00034827
AF127936.5	6.547324351	0.018122985	Up	1.85873162	Up	0.01335534
LINC00582	5.898576504	0.008764889	Up	3.2200644	Up	0.00020707
RP1-151F17.1	2.667208023	0.03133383	Up	1.93567137	Up	0.00126316
AC007386.4	7.676647949	0.0006304	Up	2.26021873	Up	0.00297033
AF127936.3	15.36066167	1.94876E−05	Up	2.15384699	Up	0.0127983
AC026202.3	3.373917889	0.035959645	Up	2.21991485	Up	0.00138382
AC062029.1	3.882550722	0.000332056	Up	1.52535853	Up	0.00023459
OLMALINC	2.522885772	0.039468434	Up	2.0138426	Up	0.00045101
RP11-325F22.2	3.93808812	1.11556E−05	Up	1.68906959	Up	0.00569426
RP11-760H22.2	9.063987853	0.00046948	Up	1.73000689	Up	0.02600696
IFNG-AS1	2.977886859	0.027079956	Up	5.42455358	Up	0.00020829
RP11-283G6.3	4.787331601	0.009367094	Up	1.84508353	Up	0.00985946
RP11-568N6.1	5.170435464	0.024819956	Up	2.450667	Up	0.01298233
MANEA-AS1	2.7658635	0.018822932	Up	1.57471363	Up	3.5726E−05
RP11-299P2.1	3.280944112	0.026855691	Up	1.50668602	Up	0.04484738
CTC-444N24.11	2.765726037	0.000419392	Up	1.82455982	Up	0.00329665
RP1-134E15.3	3.933180422	0.005893264	Up	2.9979737	Up	0.01399917
RP11-461L13.4	4.453718919	0.036662164	Up	1.92668152	Up	0.0061042
RP11-461L13.5	3.404254033	0.029779285	Up	1.73817404	Up	0.00574285
CTD-2227E11.1	6.390873704	0.001508235	Up	1.99461183	Up	0.00032089
RP11-305K5.1	3.624742583	0.009374621	Up	2.22326152	Up	0.00034388
RP11-449G16.1	3.609099306	0.008760875	Up	2.02043083	Up	0.00141473
RP11-386I14.4	2.628067724	0.013620226	Up	2.70145622	Up	0.00194904
RP11-817I4.1	2.917906091	0.038352692	Up	2.03113037	Up	0.00264876

DE lncRNAs, differentially expressed long non-coding RNAs.

**Table 4:** Overlapping downregulated DE lncRNAs in sequencing group and gene microarray group.

LncRNA	High-throughput sequencing			Gene chip		
	FoldChange	pval	Up/down	FoldChange	Regulation	p-Value
LINC01270	0.097725724	0.00101444	Down	−2.2274989	Down	0.00395318
RP11-211G3.2	0.045353226	0.000377896	Down	−1.5125294	Down	0.01326417
RP11-380G5.2	0.392340137	0.019992303	Down	−2.1632942	Down	0.00265532
GK-IT1	0.178858277	0.012872185	Down	−1.9298892	Down	0.00371626
LINC01001	0.217238636	0.016253344	Down	−1.5687952	Down	0.00227554
CTC-490G23.2	0.065551978	9.69592E−05	Down	−1.8917929	Down	0.00101286
DLEU2	0.5253068	0.034183143	Down	−1.5506011	Down	0.00542248
AC002511.2	0.012696862	0.000155467	Down	−1.5785111	Down	0.02846137
AC007278.3	0.0303423	2.5526E−09	Down	−3.975553678	Down	9.5626E−05
AC007278.2	0.031389875	3.62835E−10	Down	−4.2330109	Down	6.5352E−05
RP11-242C19.2	0.211326774	0.016530684	Down	−3.3116025	Down	0.00389127
FOXP1-IT1	0.275588862	0.00212099	Down	−1.5488362	Down	0.01581893
RP11-166N6.2	0.215448683	0.044964386	Down	−1.5142376	Down	0.02744864
LINC01513	0.194135632	0.043269027	Down	−1.5669143	Down	0.00146997
RP11-153M7.5	0.062758385	0.045685605	Down	−2.2940282	Down	0.00643776
RP11-414H23.3	0.049905469	0.020364237	Down	−2.7993945	Down	0.00843491
RP11-701P16.2	0.044580451	1.6142E−05	Down	−2.1304609	Down	0.00474296
RP11-582J16.5	0.407250523	0.034212539	Down	−1.5190196	Down	0.00768833
RP11-802E16.3	0.384142055	0.034735423	Down	−1.5642376	Down	0.00643966
RP11-561P12.5	0.077538685	0.001114693	Down	−4.5816899	Down	0.0022714
PLBD1-AS1	0.042490163	7.52577E−06	Down	−1.7824457	Down	0.01356517
RP11-256L6.3	0.055507422	6.59323E−05	Down	−2.5723843	Down	0.01459554
RP11-76E17.4	0.067009298	0.001607366	Down	−2.8377848	Down	0.00077204
FAM157C	0.101704459	1.64661E−07	Down	−1.7700266	Down	0.00098672
RP11-476D10.1	0.157138677	0.035038558	Down	−2.6928227	Down	0.00576235
RP11-399O19.9	0.167869555	0.013640249	Down	−1.5666437	Down	0.01445548
RP11-61F12.1	0.094675134	0.000171612	Down	−1.9591227	Down	0.00091905
RP11-553K8.5	0.106039326	0.018011961	Down	−1.6154496	Down	0.03076288
RP11-68I3.11	0.266542765	0.020141267	Down	−1.7929239	Down	0.03361848
KCNJ2-AS1	0.054571456	9.28109E−05	Down	−1.9156576	Down	0.01528959
RP11-434H6.6	0.200893223	0.00786083	Down	−1.5345556	Down	0.00040416
RP11-1046B16.3	0.365205861	0.041171409	Down	−1.7914715	Down	0.02484459
RP11-946L16.2	0.096298753	0.011678239	Down	−1.6241949	Down	0.0334753
RP13-516M14.10	0.212333891	0.048285393	Down	−1.5611568	Down	0.00170928
RP11-81A1.6	0.378617418	0.008858923	Down	−1.7242371	Down	0.02360958
RP11-358B23.7	0.131493534	4.55896E−05	Down	−1.6050978	Down	0.00691292
LINC00282	0.076213685	0.000253408	Down	−2.1520571	Down	0.00354177
LINC01127	0.090738782	0.002033181	Down	−2.6742428	Down	0.00272952

DE lncRNAs, differentially expressed long non-coding RNAs.

### 3.5 Functional prediction of DE lncRNAs in sequencing

**Figure 5:**
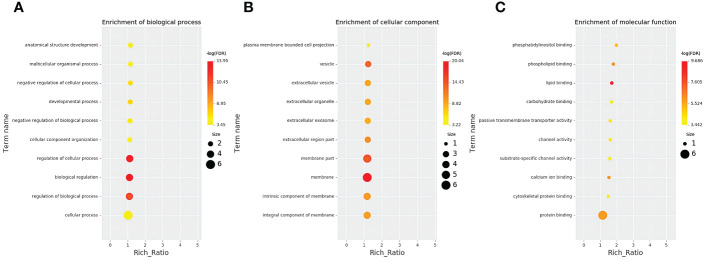
GO analysis of lncRNAs in the sequencing group. **(A)** Enrichment of biological process. **(B)** Enrichment of molecular function. **(C)** Cellular component changed mRNA-targeted ncRNAs in MM. GO, Gene Ontology; lncRNAs, long non-coding RNAs; mRNA, messenger RNA; ncRNAs, non-coding RNAs; MM, multiple myeloma.

According to co-location and co-expression of DE lncRNAs, the most significant enrichment in biological process was due to biological regulation, regulation of cellular process, and regulation of biological process; the most significant enrichment in cellular component was due to membrane, membrane part, and vesicle. Protein binding and activity were most significantly enriched in molecular function. The results are shown in **Figures 5A–C**.

**Figure 6:**
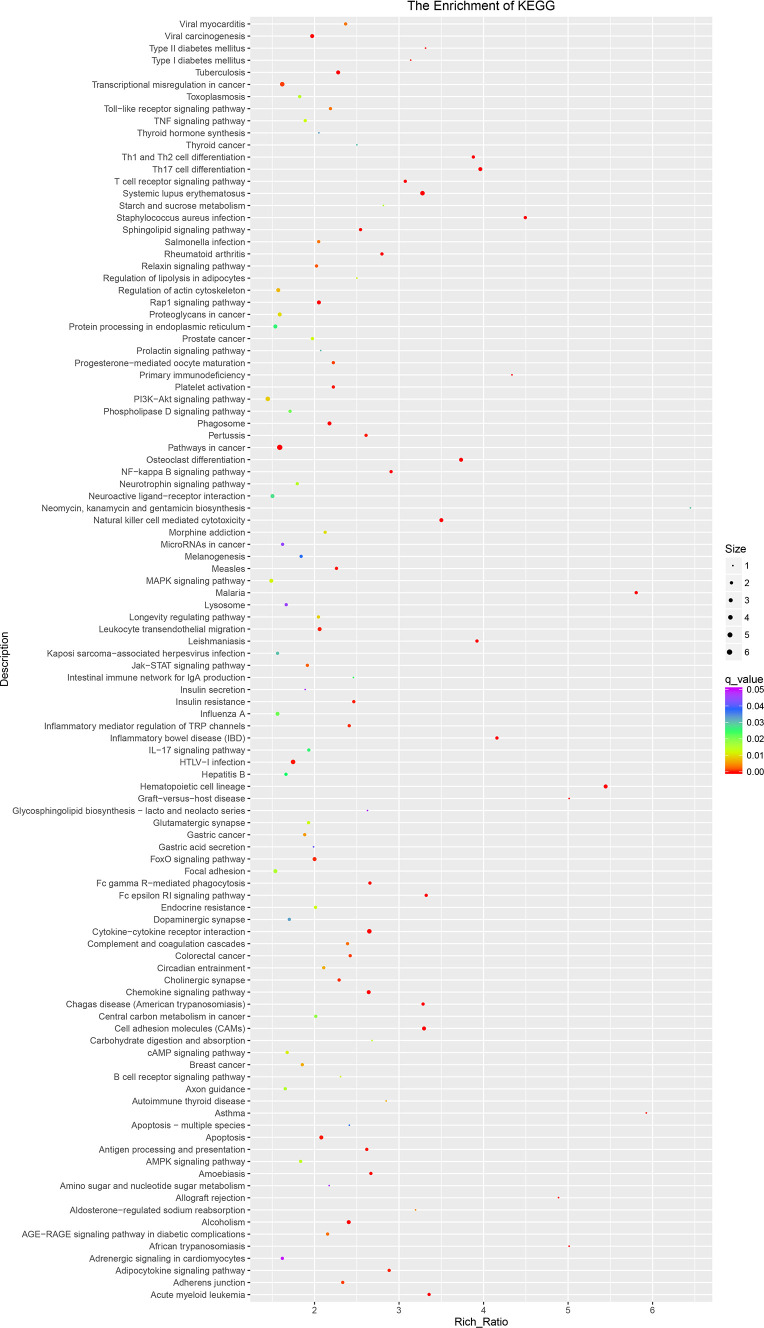
The KEGG pathway of lncRNAs in sequencing group: KEGG pathway scatterplots in the plasma cells of MM. The KO enrichment of all samples was combined, and the distribution map was drawn according to the significant Q value of the enrichment of the samples. Each point indicates the degree of enrichment of the KO entry, and the closer the color approaches red, the higher the degree of enrichment. The size of each point indicates the number of genes enriched in the KO entry. The larger the point, the more the genes are enriched in the GO entry, and vice versa. KEGG, Kyoto Encyclopedia of Genes and Genomes; lncRNAs, long non-coding RNAs; MM, multiple myeloma; KO, KEGG Orthology.

The KEGG pathway is shown in **Figure 6**; the 15 most significant enrichment pathways included osteoclast differentiation, chemokine signaling pathway, alcoholism, malaria, systemic lupus erythematosus, leishmaniasis, leukocyte transendothelial migration, inflammatory mediator regulation of TRP channels, amoebiasis, relaxin signaling pathway, viral carcinogenesis, phagosome, pertussis, Th17 cell differentiation, and apoptosis (**Figure 6**).

### 3.6 Functional prediction of DE lncRNAs in microarray

**Figure 7:**
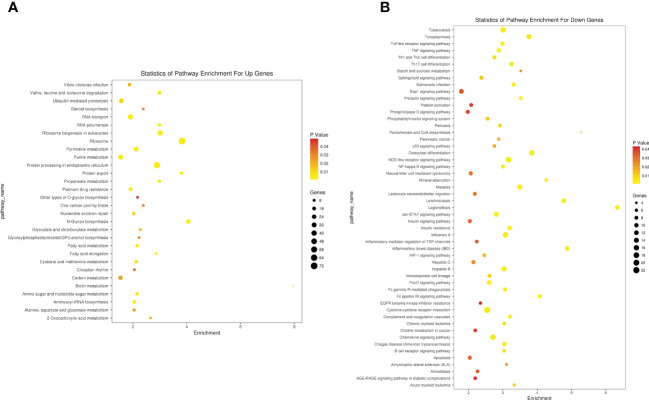
KEGG enrichment analysis of differentially expressed in microarray group. **(A)** Upregulated regulatory pathways. **(B)** Downregulated regulatory pathways. KEGG, Kyoto Encyclopedia of Genes and Genomes.

The upregulated gene functions mainly focus on protein synthesis, most of which have been confirmed to be related to cancer replication, such as rRNA processing, protein translation, and SRP- dependent co- translation proteins targeting membranes. Inhibiting neutrophil degranulation, inflammatory response, immune response, and other immune functions of killing myeloma cells while reducing hematopoietic capacity and reducing cell apoptosis is consistent with the pathogenesis of MM. KEGG analysis had similar results in gene pathway enrichment, as shown in **Figures 7A, B**.

### 3.7 Common functional prediction of DE lncRNAs in sequencing and microarray

Comparing the two lncRNA detection methods, it was found that the common pathway in the top 5 KEGG was the chemokine signaling pathway, inflammatory mediator regulation, Th17 cell differentiation, apoptosis, and NF-kappa B signaling pathway. After further analysis, the differential lncRNAs of the corresponding pathway were found. Among them, there are 20 differential genes in the inflammatory mediator regulation pathway, 18 differential genes in the Th17 cell differentiation pathway, 29 differential genes in the apoptosis pathway, and 16 differential genes in the NF- kappa B signaling pathway. The corresponding path of AC007278.2 is cytokine receptor interaction in both the sequencing group and gene microarray group.

The targeted pathway of AC007278.2 in both groups was Cytokine –Cytokine receptor interaction. AC007278.2 (sense_intronic Position:chr2:102433957-102435340:+) is Toll/interleukin-1 receptor (TIR) domain (34). The TIR homology domain is an intracellular signaling domain found in MyD88, interleukin- 1 receptor, and the Toll receptor. LncRNA AC007278.2 promotes T helper cell 1 differentiation by regulating NF-κB import into the nucleus and activating interleukin-1 receptor and interleukin-18 receptor. The KEGG targeted pathway of AC007278.2 is interleukin- 18 receptor 1 and IL1RRP. The KEGG targeted pathway of FAM157C (cRNA Position:chr16:90102271-90186204:+) has potential kinase structure characteristics; the KEGG targeted pathway of FAM157C is adenylate cyclase 4.

### 3.8 qPCR verification of lncRNA

**Figure 8:**
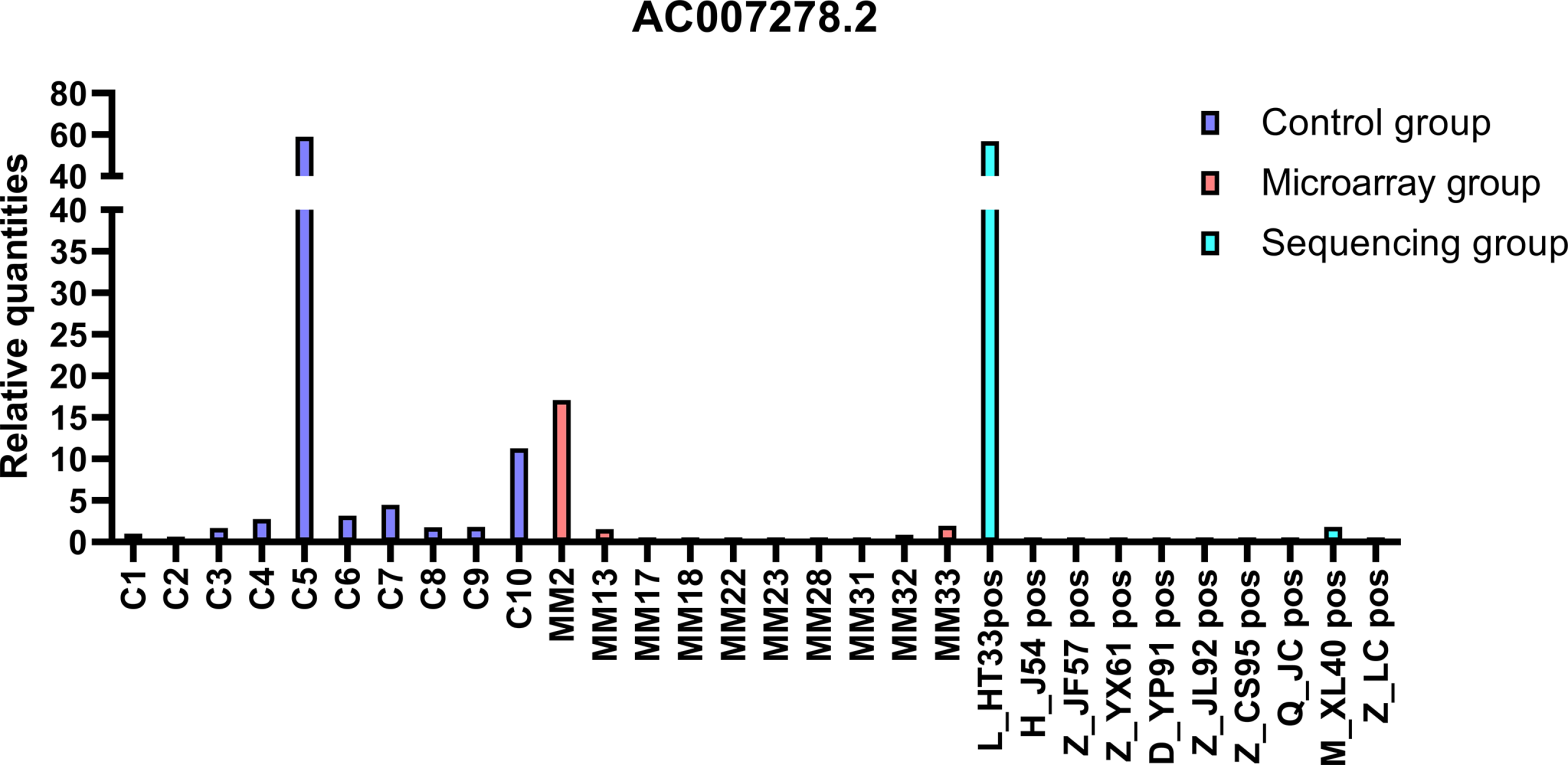
The qPCR results of AC007278.2: AC007278.2 was downregulated in sequencing group and microarray group compared with the control group. The ordinate is the relative expression of AC007278. 2.

**Figure 9:**
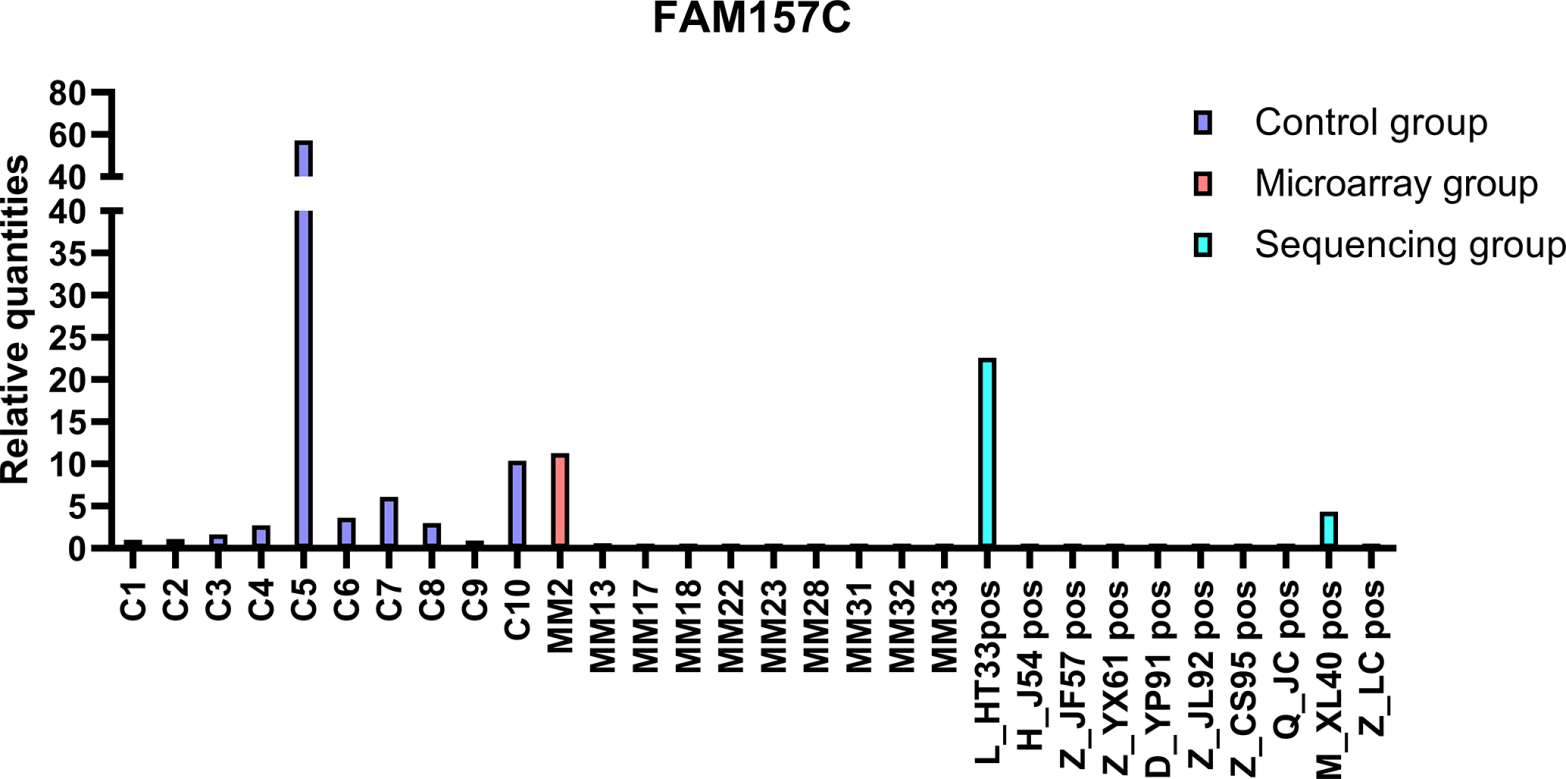
The qPCR results of FAM157C: FAM157C was downregulated in sequencing group and microarray group compared with the control group. The ordinate is the relative expression of FAM157C.

The top 20 DE lncRNAs were verified by overlapping DE lncRNAs, including AC007278. 2 and FAM157C, especially AC007278.2, which was the most differentially expressed lncRNAs in the sequencing group. The qPCR results were consistent with the sequencing results (**Figures 8**, **9**). AC007278. 2 and FAM157C were downregulated in both the sequencing group and the chip group.

### 3.9 Analysis of the regulatory network of lncRNAs (ceRNAs)

**Figure 10:**
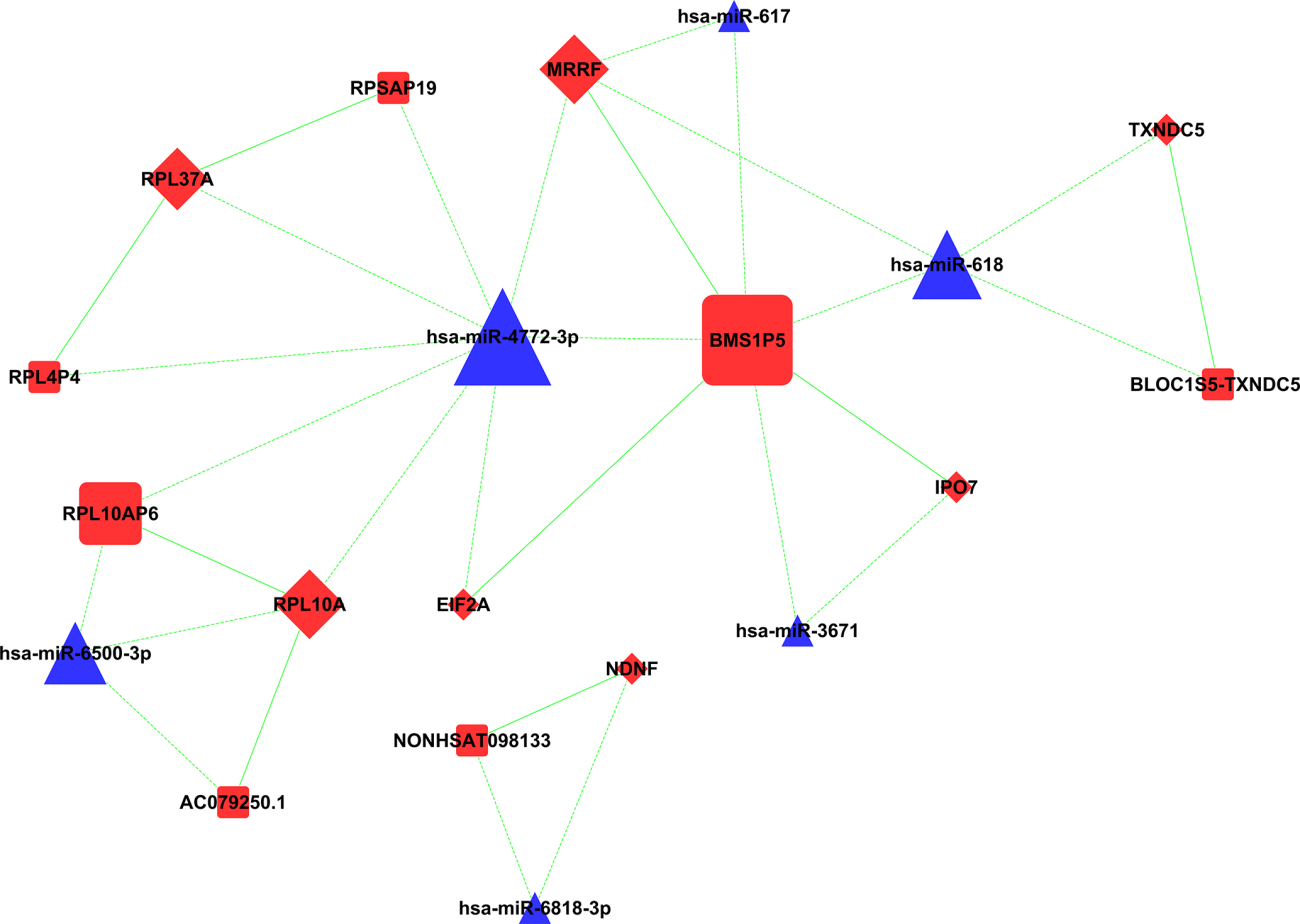
CeRNA network in microarray group: gene molecules with ceRNA relationship are shown in this network. Square, triangle, and diamond represent lncRNA, miRNA, and mRNA, respectively. ceRNA, competing endogenous RNA; lncRNA, long non-coding RNA; miRNA, microRNA; mRNA, messenger RNA.

A total of 19 known lncRNAs and 116 novel lncRNAs were speculated to constitute ceRNA with miRNAs and mRNA in the sequencing group; the proportion of novel lncRNAs was significantly higher than that of known lncRNAs. Only a small number of RNAs were shown to be involved in the ceRNA network in the microarray group, including 9 lncRNAs, 8 miRNAs, and 51 mRNAs (**Figure 10**).

Among these genes, three miRNAs constituted ceRNA of lncRNA–miRNA–mRNA in both the sequencing group and microarray group, including miR-4772-3p, miR-617, and miR-618. MiR-4772-3p was upregulated in malignant plasma cells, which constituted ceRNA with ZNF850 and novel lncRNA-MSTRG.60361 in the sequencing group. MiR-4772-3p was speculated to constitute ceRNA with BMS1P5, RPL10AP6, RPL4P4, RPSAP19, RPL12P4, and RPS13P2 in microarray. MiR-617 constituted ceRNA with novel lncRNA-MSTRG.255209, MSTRG.42167, and MSTRG.42200 in the sequencing group, while miR-617 constituted ceRNA with BMS1P5 in the microarray. MiR-618 constituted ceRNA with HIC1, MSTRG.42140, and MSTRG.42235 in the sequencing group, the pathway dominated by BTB/POZ. MiR-618 was speculated to constitute ceRNA with BLOC1S5-TXNDC5 and BMS1P5 in the microarray group.

## 4 Discussion

MM is the second most common hematological malignancy. Recently, microarrays and sequencing have become important tools for identifying treatment targets and developing personalized treatments. In our study, we detected overlapping differential expression of lncRNAs and identified their common functions through high-throughput sequencing and genetic microarray. We also investigated the ceRNA of multiple myeloma and selected two significant overlapping differentially expressed lncRNAs that could be used as potential therapeutic targets for MM.

The study found that lncRNAs were significantly dysregulated in MM cells. A total of 1,263 known lncRNAs were detected to be significantly regulated in malignant plasma cells of newly diagnosed MM (NDMM) by microarray assay, and more DE lncRNAs were found by high- throughput sequencing, in addition to 1,559 known lncRNAs. After comparison, the two-study mode has a common finding. Among them, 64 overlapping lncRNAs were found to be differentially expressed in both the sequencing group and the microarray group. Some lncRNAs up regulated in different diseases, such as IFNG-AS1 (35), AC093818.1, AF127936.5, and OLMALINC.IFNG-AS1, have been reported in many malignant tumors. Among the down regulated lncRNAs, DLEU2, LINC01270, LINC01270, GK-IT1, LINC01001, DLEU2, CHRM3-AS2, AC007278.2, PLBD1-AS1, FAM157C, KCNJ2-AS1, and LINC01127 were abnormally expressed in other diseases in previous studies. Among them, AC007278.2 and FAM157C showed more significant differences in the two groups, which may be important regulatory factors for the development of multiple myeloma. Especially, AC007278.2 is the top 1 DE known lncRNA in the sequencing group, which may become a potential therapeutic target of multiple myeloma.

The study found that lncRNAs were significantly dysregulated in MM cells. Microarray assay detected 1,263 known lncRNAs that were significantly regulated in malignant plasma cells of NDMM, and more DE lncRNAs were found by high- throughput sequencing, including 1,559 known lncRNAs. Comparison between the two methods revealed common findings, with 64 overlapping lncRNAs identified as differentially expressed in both sequencing and microarray groups. Some lncRNAs were found to be upregulated in different diseases, such as IFNG-AS1, AC093818.1, AF127936.5, and OLMALINC; others were downregulated in various diseases, such as DLEU2, LINC01270, GK-IT1, LINC01001, CHRM3-AS2, AC007278.2, PLBD1-AS1, FAM157C, and KCNJ2-AS1. Among them, AC007278.2 and FAM157C showed more significant differences in the two groups, indicating their potential role as important regulatory factors in the development of MM. Specifically, AC007278.2 was identified as the top DE known lncRNA in the sequencing group and may represent a potential therapeutic target for MM.

The comparison of the function of lncRNAs using two detection methods revealed that the top 5 KEGG pathways with commonly dysregulated lncRNAs were the chemokine signaling pathway, inflammatory mediator regulation, Th17 cell differentiation, apoptosis, and NF-kappa B signaling pathway. Moreover, lncRNAs were found to be involved in the regulation of apoptosis pathways and immune function- related pathways. The identification of these pathways and related lncRNAs provides a clearer understanding of the role of lncRNAs in MM. Further *in vivo* and *in vitro* validations will be conducted in future studies to confirm these findings.

Comparing ceRNA networks, lncRNA–miRNA–mRNA networks were speculated from the two methods. The analysis revealed that miR-4772-3p, miR-617, and miR-618 were identified as ceRNAs with lncRNAs in malignant plasma cells in both groups. Although miR-4772-3p has been less investigated in previous research on malignant tumors, it was speculated as an important miRNA of ceRNA in both detection methods, requiring further research for confirmation. Our findings also suggest that miR-618 may be involved in MM mechanisms. Previously, miR-618 was shown to play a role in tumor inhibition by targeting XIAP expression (36). In our study, miR-618 constituted ceRNA with HIC1, MSTRG.42140, and MSTRG.42235 in the sequencing group, with the pathway being dominated by BTB/POZ.

The study revealed common differentially expressed lncRNAs, functions, and ceRNA in newly diagnosed MM provided by whole-genome sequencing and whole-genome microarray. Gene chip is a closed detection system that can only detect known gene information, while sequencing is an open detection system that can find new gene data. After nearly 20 years of development, the chip platform has greatly improved quality control standards and analysis mode, enabling faster results. However, sequencing is the mainstream technology, and it is powerful in completing massive data analysis and finding more new genes. The results obtained by the two detection methods can mutually verify the expression and function of lncRNAs in MM. Co-expression of DE lncRNAs was identified, which is associated with the pathogenesis of MM, contributing to the further exploration of different biological characteristics in MM.

In conclusion, both sequencing and microarray are powerful methods for generating genome-wide information, and our comparative study suggests that the two methods produce similar association profiles, enabling the integration of both data sets. However, a limitation of our study is the limited number of relevant studies currently available. A more comprehensive study with larger sample size is needed to clarify the expression of non-coding RNA in MM and validate potential target genes, as well as to explore the underlying mechanisms.

## Data availability statement

The datasets presented in this study can be found in online repositories. The names of the repository/repositories and accession number(s) can be found in the article/**Supplementary Material**.

## Ethics statement

All participants signed the informed consent, and this study was approved by institutional ethical review board of the Chao-Yang Hospital, Capital Medical University (Ethical approval code 2016-science-84, 2019-science-118).

## Author contributions

Conceptualization: M-QL and W-MC. Data curation: YJ. Formal analysis: WG. Funding acquisition: W-MC. Project administration: LB. Resources: H-XZ. Software: YW. Supervision: LB. Validation: H-XZ. Writing—original draft: Y-QH. All authors contributed to the article and approved the submitted version.
