# Supplementary material for: Identification of aberrantly expressed lncRNAs and ceRNA networks in multiple myeloma: a combined high-throughput sequencing and microarray analysis
Source: Front Oncol. 2023 Jun 5;13:1160342. doi: 10.3389/fonc.2023.1160342 (PMC10277558; doi:10.3389/fonc.2023.1160342)
Supplement: Supplementary file 3 [file Table_3.docx]

**Detection of the change of target gene expression in human cell samples by Real Time PCR**

1. **RNA electrophoresis map**

M 1 2 3 4 5 6 7 8 9 10 11 12 13 14 15 16 17 18 19 20


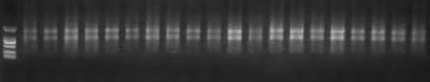


Figure 1: Lane1-20 corresponds to 20 samples in the sample list, and the sequence of electrophoresis sample names is consistent with the sample number in the sample list.

M: DNA Marker: DM2000, 1002505007501000 and 2000bp from bottom to top, of which 750bp is a bright band.

**2. Real Time PCR test results**

Standard curve of gene amplification:Take sample cDNA for 5-fold gradient dilution, and take 2 samples after dilution μL as a template, amplify with the target gene primer, and analyze the fusion curve at 60-95 ℃. The instrument automatically draws the standard curve of the corresponding target gene.


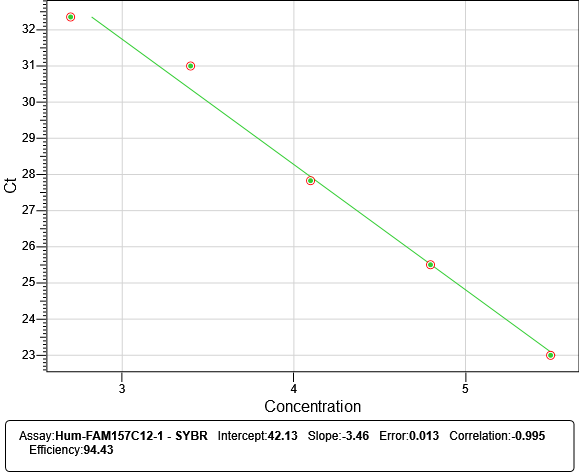

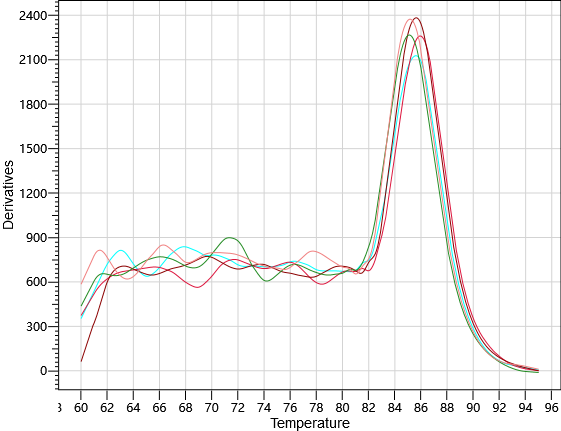


Figure 2: Standard curve and product dissolution curve of FAM157C.


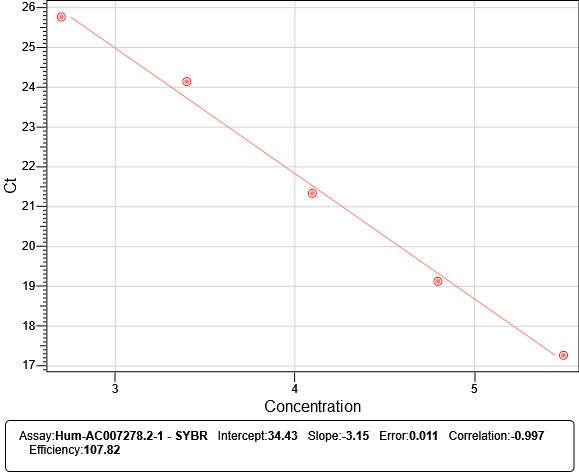

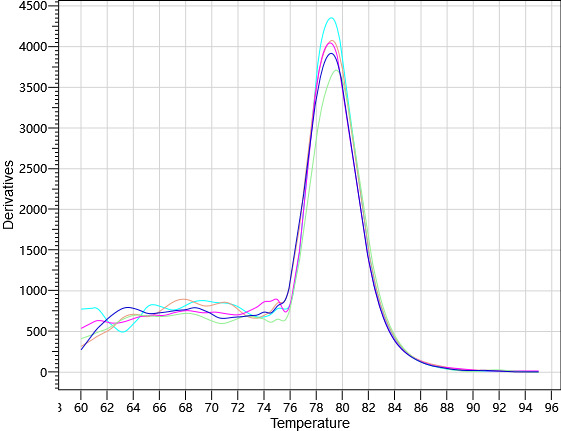


Figure 3: Standard curve and product dissolution curve of AC007278.2.

**3 Real-time amplification curve of each sample and dissolution curve of sample amplification products**


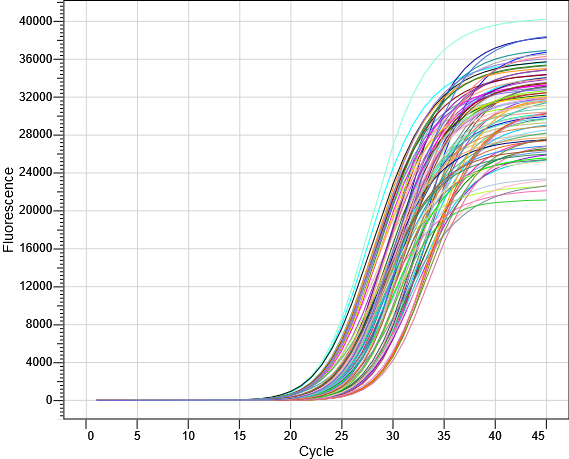

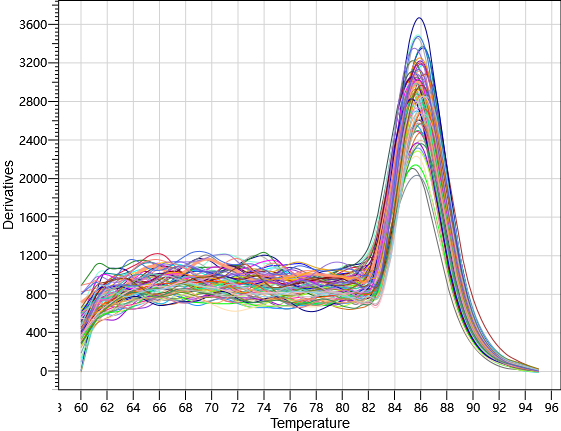


Figure 4 :Real-time amplification curve and product dissolution curve of FAM157C.


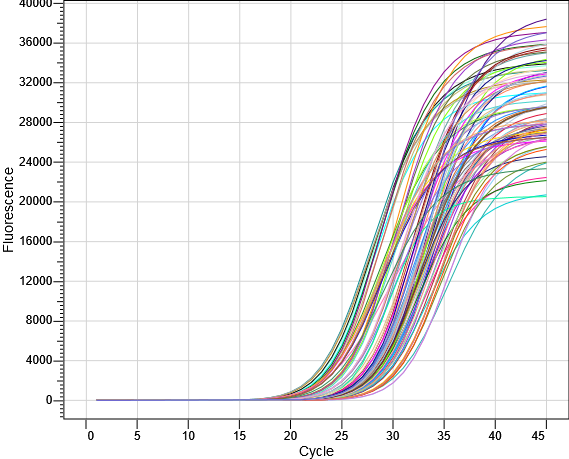

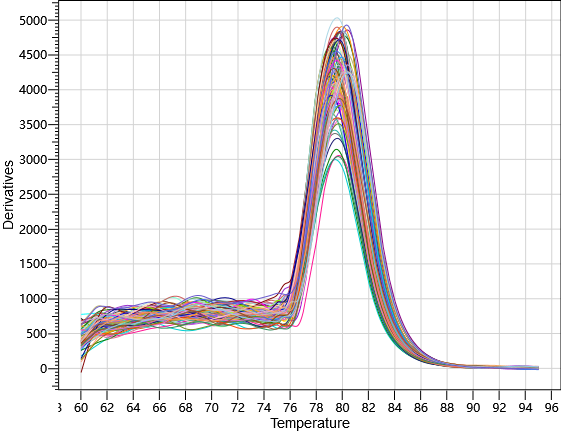


Figure 5：Real-time amplification curve and product dissolution curve of AC007278.2.


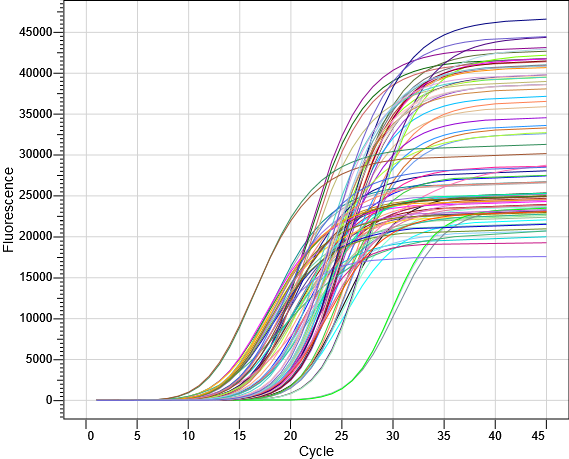

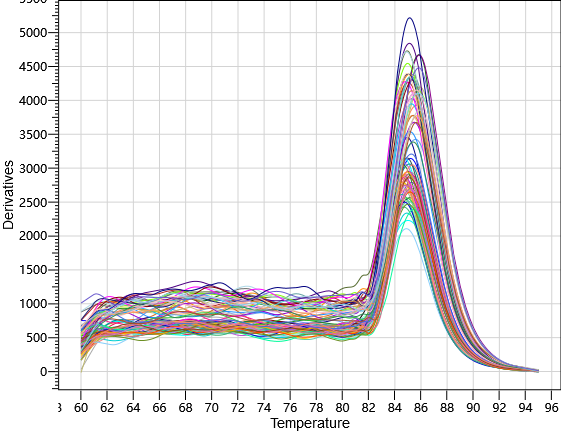


Figure 6: Real-time amplification curve and product dissolution curve of GAPDH gene of internal reference.
